# Tamoxifen administration in pregnant mice can be deleterious to both mother and embryo

**DOI:** 10.1177/0023677219856918

**Published:** 2019-06-27

**Authors:** Nikita Ved, Angela Curran, Frances Mary Ashcroft, Duncan Burnaby Sparrow

**Affiliations:** 1 Department of Physiology, Anatomy and Genetics, University of Oxford, UK; 2 Department of Biomedical Services, University of Oxford, UK

**Keywords:** 3Rs, ethics and welfare, genetics, organisms and models, rodents, experimental design, techniques, laboratory animal welfare

## Abstract

Since it was introduced 20 years ago, tamoxifen-inducible genetic recombination in vivo has become a standard tool in many fields. This technique has great utility, allowing precise temporal and spatial gene recombination mediated by expression of a Cre recombinase-oestrogen receptor hormone binding domain fusion protein. It is frequently used in developmental biology, either for accurate spatio-temporal gene deletion or for lineage-labelling. Administration of high doses of tamoxifen can rapidly induce abortion in pregnant mice but this can be partially overcome by progesterone co-administration. However, administration of tamoxifen to pregnant mice early in pregnancy may have potentially lethal effects on the mother independently of abortion, and can also severely perturb embryonic development. Despite this, only a few published studies mention this fact in passing, and standard parameters for successful or unsuccessful use of tamoxifen in pregnant mice have not been reported. Therefore, in the interests of providing a framework for more humane animal research, we describe our experiences of tamoxifen administration during early gestation in mice. These observations should assist the design of future studies in accordance with the principles of the three Rs (Replacement, Reduction and Refinement of Animals in Research).

## Résumé

Depuis son introduction il y a 20 ans, la recombinaison génétique pouvant être induite par le tamoxifène *in vivo* est devenue un outil standard dans de nombreux domaines. Cette technique a une grande utilité puisqu’elle permet une recombinaison génétique spatio- temporelle précise médiée par l'expression d’une protéine de fusion recombinase CRE avec le domaine de liaison du récepteur des œstrogènes. Elle est fréquemment utilisée en biologie du développement, que ce soit pour une délétion spatio-temporelle précise de gène ou pour l'étiquetage par lignée. L'administration de doses élevées de tamoxifène peut rapidement provoquer un avortement chez les souris gravides mais cela peut être partiellement surmonté par la co-administration de progestérone. Toutefois, indépendamment de l'avortement, l'administration de tamoxifène à des souris gravides au début de la grossesse peut avoir des effets potentiellement dangereux sur la mère voire perturber gravement le développement embryonnaire. Malgré cela, seules quelques études publiées mentionnent ce fait en passant, et aucun paramètre standard pour la réussite ou l'échec de l'utilisation du tamoxifène chez les souris gravides n'a été rapporté. Par conséquent, afin de fournir un cadre permettant une recherche animale plus humaine, nous décrivons nos expériences d'administration de tamoxifène à des souris, au début de la gestation. Ces observations devraient aider à concevoir les études futures conformément aux principes des trois R (Remplacement, Réduction et Raffinement des animaux utilisés dans la recherche).

## Abstract (de)

Seit ihrer Einführung vor 20 Jahren ist die Tamoxifen-induzierbare genetische Rekombination* in vivo* in vielen Bereichen zu einem Standardwerkzeug geworden. Diese Technik bringt großen Nutzen und ermöglicht eine präzise zeitliche und räumliche Genrekombination, die durch ein Cre-Rekombinase-Östrogen-Rezeptor-Hormon-Bindungsdomänen-Fusionsprotein vermittelt wird. Es wird häufig in der Entwicklungsbiologie eingesetzt, entweder für die genaue räumlich-zeitliche Gendeletion oder für die Zelllinien-Kennzeichnung. Die Verabreichung von Tamoxifen in hohen Dosen kann bei trächtigen Mäusen schnell zu einem Abort führen, was jedoch durch die simultane Verabreichung von Progesteron teilweise verhindert werden kann. Die Verabreichung von Tamoxifen an trächtige Mäuse zu Beginn der Trächtigkeit kann jedoch unabhängig von einem Abort potenziell tödliche Auswirkungen auf die Mutter haben und kann auch die embryonale Entwicklung stark beeinträchtigen. Dennoch erwähnen nur wenige veröffentlichte Studien diese Tatsache – und auch dann nur nebenbei – und es liegen keine Standardparameter für einen erfolgreichen oder erfolglosen Einsatz von Tamoxifen bei trächtigen Mäusen vor. Daher beschreiben wir zur Schaffung eines Rahmens für eine humanere Tierforschung unsere Erfahrungen mit der Gabe von Tamoxifen während der frühen Trächtigkeit bei Mäusen. Diese Beobachtungen sollten die Gestaltung zukünftiger Studien in Übereinstimmung mit den Prinzipien der drei Rs (Replacement, Reduction and Refinement of Animals in Research) unterstützen.

## Resumen

Dese su introducción hace 20 años, la recombinación genética inducible con tamoxifeno *in vivo* se ha convertido en el una herramienta estándar en muchos campos. Esta técnica tiene gran utilidad ya que permite una recombinación de genes espacial y temporal precisa mediante la expresión de una fusión de la proteína Cre recombinase y el dominio vinculante hormonal de la proteína del receptor de estrógeno. A menudo se utiliza en biología de desarrollo, ya sea para la eliminación de genes espacio temporales precisos como para la etiquetación de linajes. La administración de altas dosis de tamoxifeno puede inducir rápidamente el aborto en ratones embarazados pero esto puede superarse parcialmente con una coadministración de progesterona. Sin embargo, la administración de tamoxifeno en ratones a principios del embarazo pueden tener efectos letales en la madre independientemente del aborto y también puede perjudicar gravemente el desarrollo del embrión. A pesar de esto, únicamente unos cuantos estudios publicados mencionan este hecho de pasada, y los parámetros estándar para un uso satisfactorio o insatisfactorio de tamoxifeno en ratones embarazados no han sido publicados. Por tanto, a fin de ofrecer un marco para una investigación con animales más ética, describimos nuestra experiencia con la administración de tamoxifeno durante los inicios de gestación en ratones. Estas observaciones deberían ayudar a diseñar estudios futuros de acuerdo con los principios de las tres R (Reemplazo, Reducción y Refinamiento de animales de investigación).

**Figure 1.**
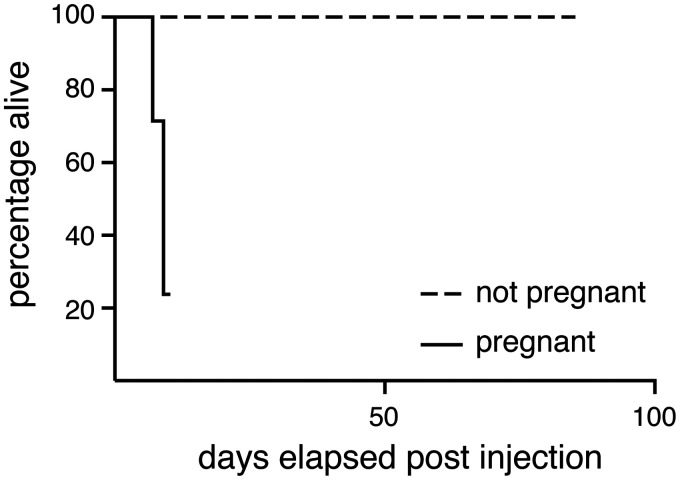
Tamoxifen-injected pregnant mice started showing signs of severe distress 5–7 days post tamoxifen injection and were euthanized as a humane end-point. By contrast, tamoxifen-injected non-pregnant females showed no effects for at least 100 days post-injection.

Six female mice of the Gt(ROSA)26Sor^tm1(Kcnj11^*^V59M)Fmas^ mouse strain^1^ were mated with male C57BL6/J mice (Charles River Laboratories, Margate, UK). Five to seven days after plugging, the pregnant females were injected subcutaneously into the scruff on one occasion with a single dose of 10–200 mg/kg tamoxifen (Sigma-Aldrich, Gillingham, UK) dissolved in corn oil (Sigma-Aldrich, Gillingham, UK; see Table 1 for study design). Tamoxifen is metabolized in vivo to 4-hydroxy-tamoxifen, which acts as an antagonist of the oestrogen receptor. In research, it can also be used to conditionally activate the Cre-ERT2 fusion protein to cause conditional genomic recombination. In this study, the mice did not carry a Cre-ERT2 allele, so tamoxifen treatment would have no effect on the maternal genome. These mice were derived from a colony that had been backcrossed for more than 10 generations onto the C57BL/6J background. They were housed in a specific-pathogen-free facility free from the major rodent pathogens except *Helicobacter hepaticus*, with a 12:12 h light:dark cycle, at 19–23℃, 55% ± 10% humidity, in individually-ventilated cages (Tecniplast UK Ltd, Rushden, UK) containing Grade 4 Aspen Chip bedding (Datesand Ltd, Manchester, UK), cardboard tunnels and Sizzle Pet nesting material (LBS Biotechnology, Horley, UK), with free access to Teklad 2916 mouse chow (Envigo, Huntingdon, UK) and tap water. Bedding was changed fortnightly, and animals were assessed daily for welfare. Injections took place in the morning in a flow hood, and mice were immediately returned to their home cage after injection. Unexpectedly, all (6/6) mice developed severe intrauterine haemorrhage 7–9 days post-injection, and 3/6 had to be euthanized as a humane endpoint for ethical reasons 1–3 days prior to the planned embryo harvest date on day E15.5 (Figure 1 and Table 1). The three animals euthanized early had been dosed with 200 mg/kg on E5.5, 100 mg/kg on E7.5 and 10 mg/kg on E5.5, respectively. This effect was apparent at a range of doses commonly used in the literature to activate the tamoxifen-inducible Cre recombinase (10–200 mg/kg). We compared the effects of unmated mice of the same strain and genotype injected subcutaneously with a single dose of 200 mg/kg tamoxifen. These mice were controls from another study. All control mice (104/104) survived without any deleterious effects up to 14 weeks post injection (Figure 1). This was highly statistically significant (*p* < 0.0001, two-tailed Fisher’s exact test comparing 6/6 injected pregnant mice with 0/104 injected non-pregnant mice). There is some evidence suggesting that tamoxifen dosage by oral gavage may be less toxic to pregnant mice.^2^ We therefore treated a further two pregnant mice with 100 mg/kg tamoxifen by oral gavage on E5.5 or E7.5. Both mice also suffered intrauterine haemorrhage 8–9 days after treatment, with one of these being found dead the day before the planned embryo harvest date of E15.5 (*p* = 0.0002, two-tailed Fisher’s exact test comparing 2/2 gavaged mice with 0/104 control injected mice). Therefore, we conclude that even tamoxifen administered via oral gavage might be indirectly toxic to pregnant mice if given early in pregnancy.

**Table 1.** Deleterious effects including intrauterine haemorrhage in the mother and morphological defects in the embryo were seen in the majority of mice, irrespective of tamoxifen dose. Morphological defects included: necrotic embryos, pericardial oedema (PCO), cranial neural tube defects (cNTD) such as exencephaly, craniofacial defects (CFD) including cleft palate, subcutaneous oedema (SCO) and ophthalmic defects (Oph) including microphthalmia or anophthalmia.

Mouse number	Tamoxifen dose (mg/kg)	Administration route	Day treated	Day euthanized	Uterine haemorrhage	Embryo phenotype		Embryonic defects
						Normal	Abnormal	
1	200	Subcutaneous	E5.5	E15.5	Severe	0	0	Litter resorbed
2	200	Subcutaneous	E5.5	E12.5 (early)	Severe	0	9	Necrotic, PCO, cNTD, CFD
3	100	Subcutaneous	E7.5	E14.5 (early)	Severe	0	8	PCO, CFD
4	50	Subcutaneous	E7.5	E15.5	Mild	9	1	Subcutaneous haemorrhage
5	50	Subcutaneous	E5.5	E15.5	Severe	0	6	Necrotic, PCO, cNTD, CFD
6	10	Subcutaneous	E5.5	E14.5	Severe	o	10	Necrotic, SCO, Oph
7	100	Gavage	E7.5	E15.5	Mild	6	1	Subcutaneous haemorrhage
8	100	Gavage	E5.5	E14.5 (found dead)	Severe	0	7	Necrotic, PCO, cNTD, CFD
9	0	Subcutaneous	E7.5	E15.5	None	5	0	N/A
10	0	Subcutaneous	E5.5	E15.5	None	8	0	N/A
11	0	Subcutaneous	E5.5	E15.5	None	9	0	N/A

An extensive search of the literature for other examples of maternal tamoxifen toxicity found only a single specific reference to intrauterine haemorrhage resulting from tamoxifen administration.^2^ In this case it occurred in 4/5 mice gavaged with 120 mg/kg on E6.5, haemorrhage was only apparent 12 days post-injection, and there were no apparent effects on the embryos. All other studies focused on reduced litter survival after tamoxifen administration, but not maternal health.^3–6^ In addition to maternal toxicity, we also found that maternal tamoxifen treatment had a teratogenic effect on embryos. We collected all embryos from the six subcutaneously injected and two gavaged mothers. The majority of these embryos showed abnormal development (42/57) compared with controls from three litters of mothers injected with corn oil alone (0/22, *p* < 0.0001, two-tailed Fisher’s exact test). A wide range of defects were observed, including pericardial and subcutaneous oedema, craniofacial defects, micro-ophthalmia and necrosis. In addition, one litter was completely resorbed (see Table 1). Interestingly, the two litters from mothers with mild uterine haemorrhage had the lowest rate of abnormal embryos. None of these embryos carried a Cre recombinase allele, and therefore we conclude that the observed embryonic defects were either a direct result of tamoxifen exposure or an indirect consequence of maternal uterine dysfunction.

Clinically, it is well known that tamoxifen therapy for the treatment of early-stage hormone-sensitive breast cancer in humans is contraindicated in pregnancy. AstraZeneca reported congenital anomalies in live births from mothers undergoing tamoxifen therapy during pregnancy, particularly when administered in the first trimester.^7^ Tamoxifen administration in vivo during pregnancy has been linked to impaired placental development and vascular dysfunction resulting in increased embryonic anomalies and embryonic death in rat.^8^ In summary, we believe that although tamoxifen administration during mouse pregnancy is a useful tool for precision alteration of gene expression in the study of mouse embryonic development, it can be harmful to the pregnant mouse if administered too early in gestation. Therefore, we suggest that to comply with the principles of the three Rs,^9^ future studies using tamoxifen administration to female mice early in pregnancy should proceed with caution.
